# Bayesian brain computing and the free-energy principle: an interview with Karl Friston

**DOI:** 10.1093/nsr/nwae025

**Published:** 2024-01-17

**Authors:** Wenlian Lu

**Affiliations:** Wenlian Lu is a Professor at the Institute of Science and Technology for Brain-Inspired Intelligence of Fudan University, Shanghai, China

## Abstract

The free-energy principle entails the Bayesian brain hypothesis that can be implemented by many schemes considered in this field. The combination of multimodal brain imaging and free-energy minimization has shown promise in unraveling complex brain dynamics and understanding the interactions among distinct brain regions. The Bayesian mechanics of brain computing gives a unique route to understanding authentic (neuromimetic) intelligence and, more importantly, points towards the development of brain-inspired intelligence.

NSR spoke to a leading theoretical neuroscientist and authority on brain imaging—Karl Friston, the inventor of statistical parametric mapping, voxel-based morphometry and dynamic causal modeling. Friston is also known for his contributions to theoretical biology in the form of the free-energy principle and applications such as active inference. Friston is currently the Scientific Director of the Wellcome Trust Centre for Neuroimaging, Professor of Neuroscience at Queen Square Institute of Neurology, University College London and Honorary Consultant at The National Hospital for Neurology and Neurosurgery, UK.

## Teaser

NSR spoke to a leading theoretical neuroscientist and authority on brain imaging - Karl Friston. The Bayesian mechanics of brain computing gives a unique route to understanding authentic (neuromimetic) intelligence, and more importantly, pointing towards the development of brain-inspired intelligence.

## FUNDAMENTAL PRINCIPLES OF BRAIN COMPUTING

***NSR:*** What do you think the fundamental principle of brain computing is?

***Friston:*** The fundamental principle has several names. Perhaps the neatest and most intuitive is self-evidencing. Self-evidencing refers to the imperative—for perception, cognition and action—to maximize (i.e. gather) evidence for the brain's generative (a.k.a. world) model of the sensed world. Technically, this can be articulated in terms of approximate Bayesian inference as the free-energy principle. The free-energy principle is based upon the physics of open (random dynamical) systems. It suggests that any entity with persistent characteristics can be described as self-evidencing. Practically, to simulate brain function under this principle, one would appeal to belief propagation or variational message passing on a factor graph, which subsumes the generative model and (Bayesian belief) updating scheme. Because this belief-based account is quintessentially enactive, this first principles approach suggests that the brain engages in planning as inference and subsequent action selection, under the inferred plans.

***NSR:*** Why do you think that the brain conducts Bayesian computing and what are the neurophysiological foundations of the free-energy principle?

***Friston:*** Bayesian computing is an apt way to describe brain function because this description applies to any self-organizing system that can be individuated from its environment or world (via a Markov blanket). This universal description—in terms of a Bayesian mechanics—rests upon a generative model entailed by the entity in question.

**Figure 1:**
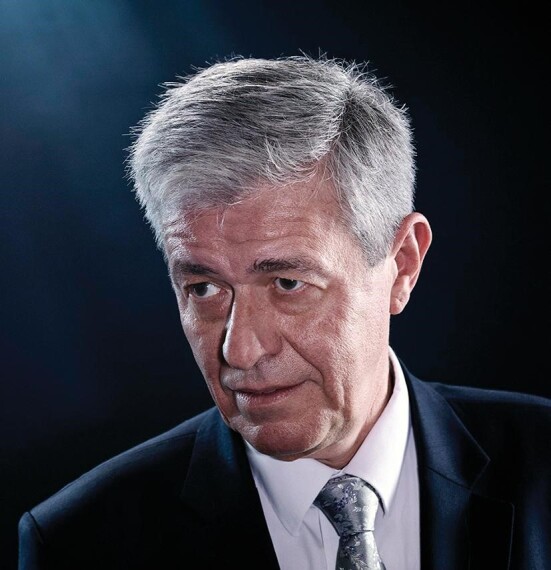
Professor Karl Friston is a leading theoretical neuroscientist and authority on brain imaging *(Courtesy of Prof. Friston)*.

Our brains probably entail the deepest, most expressive generative models in our knowable world. Here, ‘deep’ refers to hierarchical depth. This speaks to the first foundation of the free-energy principle, as applied to the brain. Namely, the functional architecture and connectivity of the brain are consistent with a deep generative model, in terms of its sparse connectivity and hierarchical organization. In terms of neurophysiology per se, the dynamics of belief propagation and variational message passing

Self-evidencing A given hypothesis *H* explains some (observable) evidence *E* and, by doing so, it provides evidence for itself [1]. That is, *E* becomes evidence for *H* to the extent that *H* explains *E*. In these cases, the fact that the evidence is available is an indispensable part of the evidential basis for *H*. Carl Hempel [2] thus described *H* as a self-evidencing explanation when the ‘information or assumption that [*E* occurs] forms an indispensable part of the only available evidential support for *H*’.

provide a compelling explanation for many aspects of synaptic processing and plasticity, ranging from functional asymmetries in forward and backward connections to neuronal dynamics.

There are many examples of these structural and dynamical foundations: they can be divided into different timescales over which Bayesian belief updating proceeds. For example, perceptual inference can be regarded as inferring latent states of the world. Procedural learning can be read as updating beliefs about generative model parameters, by changing connection strengths via experience or activity-dependent plasticity. Structure learning—at a neurodevelopmental and evolutionary timescale—can be cast in terms of Bayesian model selection, namely the selection of structures and morphologies that have the greatest model evidence or marginal likelihood.

**Figure 2:**
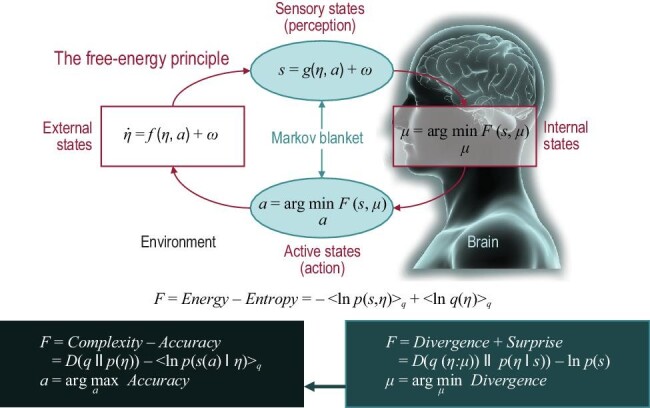
The free-energy principle [3]. (Upper Panel) Schematic of the quantities that define free energy. (Lower Panel) Alternative expressions for the free energy illustrating what its minimization entails.

## MULTISCALE MODELING OF MULTIMODAL BRAIN IMAGING

***NSR:*** Do you think neuroimaging data of the human brain, given their resolution and indirectness in terms of measuring brain functions, can (or to what degree) characterize human intelligence?

***Friston:*** Yes. One could argue that neuroimaging—of one form or another—is the only way to infer the nature of human intelligence. This follows from the fact that, to be an intelligent or sentient entity, one has to possess a Markov blanket that individuates you from everything else. Crucially, as an observer of a human brain, you can never measure directly what is inside the Markov blanket that envelops another person's brain. This means that you can only take indirect measurements, i.e. by trying to look underneath the Markov blanket, using hemodynamics or electromagnetic neuroimaging.

This means we can only infer the functional form of belief updating and requisite generative model from neuroimaging measurements. In other words, if one reads intelligence as self-evidencing, then understanding intelligence is to understand the generative model that underwrites perception, planning and action. In one sense, all imaging neuroscience speaks to this endeavor—and, in a translational setting, an understanding of the particular generative models (and implicit priors) that lead to abnormal behavior and psychopathology.

**Figure 3:**
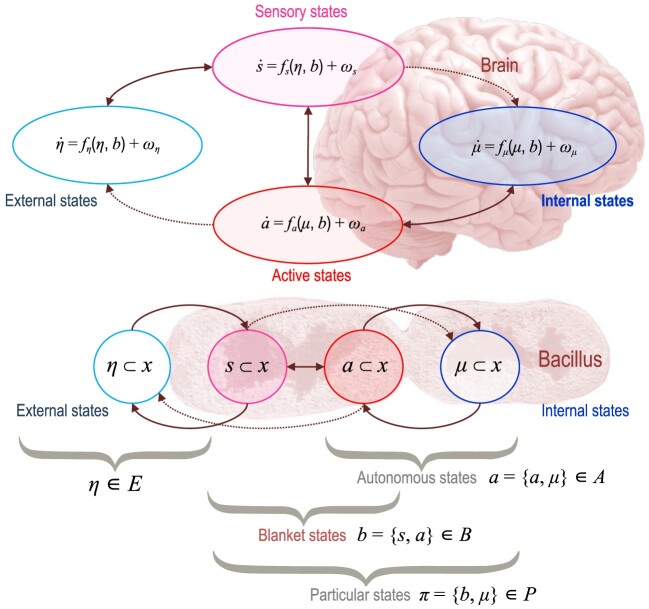
Markov blankets [4]. This influence diagram illustrates the partition of states into ‘internal’ states and hidden or ‘external’ states that are separated by a Markov blanket—comprising ‘sensory’ and ‘active’ states. The upper panel shows this partition as it would be applied to action and perception in a brain. The lower panel shows the same dependencies but rearranged so that the internal states are associated with the intracellular states of a bacillus, where the sensory states become the surface states or cell membrane overlying active states (e.g. the actin filaments of the cytoskeleton).

***NSR:*** How can we describe the different scales of multimodal brain data—from microscopic, mesoscopic to macroscopic scales—towards understanding human brain intelligence and do you think there are any neurophysiological principles here?

***Friston:*** Yes, I think there are some deep principles that link these scales. Understanding intelligence as self-evidencing requires an understanding of the generative model entailed by sentient or intelligent artifacts, such as ourselves. This necessarily involves a separation of temporal scales and, crucially, the way in which one scale contextualizes—and is informed by—the scale below (and above). The ensuing scale-invariant application of the free-energy principle has some nice consequences.

For example, it means that slow updating of model parameters both affects, and is affected by, fast updates to neuronal activity. Physiologically, this means that neuronal responses—evoked or induced by sensory exchange with the environment—depend upon synaptic connectivity. However, synaptic activity depends upon neuronal responses, in the form of activity-dependent plasticity. In a similar way, the structure and connectomics of brains affect, and are affected by, synaptic plasticity, in the sense that, for synaptic connections to exist, they have to be part of a connectome. Similarly, microscopic structure depends upon the persistence or regression of any given synaptic connection. Another example of scale-invariant or multiscale aspects of intelligence rests upon the increase in spatial scales. A nice example of this is the link between the activity of individual, spiking neurons and population activity. From the perspective of self-evidencing—under particular (continuous state-space) generative models—one can regard a population of neurons as performing exact Bayesian inference approximately, using sampling. Alternatively, one can regard a neuronal population as performing approximate Bayesian inference, exactly. In short, a multiscale approach (i.e. multiscale, multimodal measurement) is essential in providing a complete picture of intelligence, read as self-evidencing over scales.

## PROSPECTIVES FROM BRAIN COMPUTING TO ARTIFICIAL INTELLIGENCE

***NSR:*** What are the state-of-the-art areas related to brain computing and brain-inspired intelligence, and what do you think the most important issues are in these areas?

***Friston:*** State-of-the-art biomimetic computing is, mathematically, belief propagation or variational message passing on factor graphs with deep or hierarchical structure. At present, this kind of computing is simulated on von Neumann architectures. One might imagine that (in a few years’ time) state-of-the-art will elude the von Neumann bottleneck and turn to in-memory processing, using reactive message passing (along the lines of the actor model in computer science).

This can also be compared to the notion of mortal computing (i.e. natural computing), under the constraint that the computation just is the exchange of messages that underwrite self-evidencing. The closer this kind of mortal or neuromimetic variational message passing comes to neuronal dynamics on brain structures, the closer we will get to state-of-the-art brain computing in artificial intelligence (AI). Put another way, the closer the generative models used for belief propagation and variational message passing are to our own generative models, the more AI will come to resemble natural intelligence and, therefore, become state-of-the-art.

***NSR:*** How can these principles of knowledge about the brain intelligence inspire AI theory and technologies and what are the fundamental routines in your opinion?

***Friston:*** The dénouement of the above arguments, for AI, are straightforward. The direction of travel should be towards the selection—or structure learning—of generative models apt for exchange with other artifacts and humans; in an ecosystem of distributed intelligence or self-evidencing. This will require a kind of in-memory processing that can be read as variational message passing on a graph. This graph may be on a computer chip, the World Wide Web or in your brain. However, the principles that underwrite the scheduling and structuring of the requisite message passing remain the same at every scale.
